# Surgery

**Published:** 1838-07

**Authors:** 


*Zeitschrift für die gesammte Medicin.* Band vi. Heft i.

### SURGERY.

### *Questionable Cause of a fatally terminating Case of Phlebitis, after Venesection.* By Dr. Spörer.

A single woman, ætat. 32, of fresh complexion, was attacked ten days before her admission into the hospital, with rigors, dry cough, and pain in the head and hepatic region: this was followed by a jaundiced aspect, and, on the night previous to admission, by delirium. When admitted, March 17, 1834, the pulse was full, incompressible, and 100; skin yellow, hot and arid, and the tongue brownish red, dry, and hard: the bowels had not been relieved for two days. A mild aperient was ordered, to be taken every two hours. The succeeding night was spent in great restlessness and delirium; pain in the head and right hypochondrium continued with increased fever: the veins of the extremities were turgid. Venesection was now performed in the right arm to about ℥x.; and sinapisms were applied to the epigastrium and calves of the legs: an anodyne mixture was also ordered, to moderate the purging which had now set in. The blood which had been withdrawn was coated with a greenish-yellow crust, and presented much green serum. On the two following days (19th and 20th), the symptoms were mitigated in some respects: the pulse sunk to ninety-five and became softer, and the tenderness of the hypochondrium had decreased; but the pain in the head was very severe, the tongue continued dry and the skin had assumed a deeper tinge of yellow. On the 21st there was increased febrile excitement with delirium; the pain in the hypochondrium had returned in full force, and the evacuations from the bowels were frequent, watery, and fetid. It was at this time (the third day after venesection) that an erysipelatous blush appeared at the bend of the elbow, surrounding the cicatrix of the lancet puncture; and, extending upwards in the course of the brachial vein, a distinct, dark streak was observed: further, there were evidences of decreasing power, in a weak, quick and small pulse, and tremulous hand. The remedies now employed were calomel. gr. i., opii gr. $\frac{1}{4}$, ter die, and small doses of acid. This obtained some temporary mitigation of symptoms, but, in spite of further treatment, the patient sunk, and died on the 25th.

Sixteen hours after death, the inflamed arm was examined. Some tea-spoonfuls of fetid pus flowed from the gaping wound where venesection had been performed. The median vein, and the brachial as far as the clavicle, were red, inflamed, and elastic like an artery. The neurilema of the median nerve was intimately adherent to the vein, and, in the vicinity of the puncture, inflamed and of a florid appearance; but the nerve presented no evidence of having been injured by the lancet. A further examination of the cavities was unfortunately not permitted; a point much to be regretted as detracting materially from the interest of the case.

Dr. Spörer remarks that, in this case, it appears very questionable whether the phlebitis could have resulted, as a secondary consequence, from injury of the nervous sheath: but he is far more inclined to regard as the cause, an inflammatory disposition on the part of the veins, in hepatitis, induced by the irritation of bile circulating with the blood. Of three cases of phlebitis following venesection, which he has treated, two did well under the free application of leeches and repeated drastic purges. In one of these, acute inflammation of the liver was likewise present; and in all three the hepatic system suffered in a greater or less degree.

[The neglect of a very simple precaution in the mode of opening a vein is, probably, a very frequent and overlooked cause of phlebitis: we allude to the propriety of making the section in a longitudinal direction, instead of either transversely or obliquely. By this plan the adhesive process is most readily promoted,—a point of much importance, as will be readily admitted, when we consider the risk which may attend the reparative process by suppuration, which is then the only alternative that remains. The facts here noticed have been experimentally verified by an intelligent French surgeon who communicated to us the result of his investigations. The practical importance, if not the novelty of this suggestion, may be deemed sufficient apology for the introduction of this note.]

*Zeitschrift für die gesammte Medicin.* Band vi. Heft ii.

---

*Luxation of the scapular Extremity of the Clavicle, downwards.*
By Dr. TOURNEL.

THIS case is interesting, as being, apparently, with one exception, the first of the kind hitherto detailed; and as being one, also, the possibility of which has been questioned.

A soldier was thrown from his horse. The horse fell on its rider, and, in recovering itself, placed its foot on the front of his left shoulder, where was an ecchymosis of almost the exact shape of the horse's shoe The pressure separated the scapula backwards. The clavicle remained attached to the sternum; but, its superior and inferior and coraco-clavicular ligaments having been torn, its external extremity slipped from its articular surface beneath the acromion. The accident was regarded at first as a dislocation of the humerus, but the true nature of it was ascertained by an examination in the following manner: The summit of the shoulder was grasped with one hand, resting on the acromion, whilst, with the other hand, it was ascertained, by various motions, that the axis of the humerus was in its ordinary direction. There was no bony projection in the axilla. The left arm was somewhat longer than the right; the elbow and all the rest of the limb were in contact with the lateral part of the trunk. Voluntary movements, and especially upwards, were impracticable: communicated movements were easy, and unattended with pain. The shoulder had lost its rounded form, and there was a depression outwards, beneath the acromion. The shoulder presented, in addition, two projections; one internal and superior, formed by the acromion; the other external and inferior, formed by the external extremity of the clavicle. There was no numbness or pain of the fingers. The summit of the left shoulder was much nearer the sternum than that of the right; and, when the finger was passed along the spine of the scapula from behind forwards, as far as its acromial extremity, it was not stopped by the clavicle. This had been perfectly recognized; and it disappeared, together with the sub-acromial depression, when, the knee having been placed between the two shoulders, they were both drawn backwards: but, when this traction was discontinued, the projection, formed by the external extremity of the clavicle, and the depression, were reproduced. The association of all these symptoms leaves no room to doubt that this accident was a dislocation of the scapular extremity of the clavicle, downwards. The reduction was easily effected by placing the knee against the vertebral column, and drawing the shoulders backwards. A cushion was then placed, and retained by bandages in the axilla. The arm pressing upon the cushion was kept applied to the trunk upwards and backwards, by Desault's bandage for fracture of the clavicle. Spirituous lotions were applied to the shoulder. The fore-arm was placed in a sling, and the whole kept in position by a bandage passing round the body. The impatience of the soldier required the removal of this apparatus. Instead of it was substituted that of M. Flamant, which has the advantage of leaving the injured part uncovered. This consists of a grooved bag,* to the angles of which are sewn two rollers, and of a pad, which is placed, as above, in the axilla. The arm being placed in the bag so that the elbow corresponded to its middle angle, the roller, sewn to its anterior angle, was passed over the middle and dorsal part of the fore-arm, and continued in front of the chest. The other was continued over the back part of the arm, and crossed the former over a thick compress placed upon the uninjured shoulder. The rollers were continued in these directions for two or three turns, crossing one another over the uninjured shoulder, and beneath the elbow of the opposite side. The remainder of the rollers was then passed round the trunk, in order to fix the arm. At the elbow, the bandage was kept in its situation by four tapes, two of which were sewn to the inner side, and two to the outer side of the sac in which the elbow rested. The whole apparatus was covered by a bandage of the trunk, and the scapulary of this bandage was employed to keep resolutive compresses applied to the injured shoulder. Notwithstanding his impatience, the soldier perfectly recovered, after thirty-two days' treatment. The first use which he made of his arm was to severely castigate his horse. He remains in his regiment, and experiences neither pain in the shoulder nor difficulty in the movements of the arm.

In the "Ephémérides Nat. Cur.," is an account of a similar displacement. In both cases the cause was violence from above, and directly upon the scapular extremity of the clavicle.

*Archives Générales de Médecine. Décembre,* 1837.

---

*On the Excision of Ulcers, which succeed to small Subcutaneous Abscesses of a Syphilitic and Scrophulous Nature.* By M. Bonnet.

The syphilitic ulcers here spoken of are preceded by an accumulation of pus, which elevates the skin without changing its colour. Subsequently the skin acquires a coppery hue, becomes thin, more elevated, and ulcerates at its centre. A small quantity of pus escapes, and the ulcer increases in size until it has destroyed all the detached skin. It is rounded, and its base is of a greyish colour. As these ulcers are numerous, all their stages may be examined in the same individual. They, of course, require a constitutional treatment; but if, after this treatment, they continue stationary, and refuse to heal under the employment of local means, they should be converted into simple wounds, either by cauterizing them, or, what is better, by excision. Cases are related in proof of the advantage of excising ulcers of this kind, under the circumstances above mentioned.

The author appears to have applied the same treatment to ulcers of a scrophulous character; the object being always to remove the whole ulcer, its base and borders. A great number of cases is said to have been thus treated, and with results which

* Saç en forme de gouttière.

greatly recommend the treatment; the process of healing being very rapid when the diseased surface has been entirely removed.

*Archives Générales de Médecine. Décembre*, 1837.

---

*New Method of reducing Dislocations of the Os Humeri.* By M. MALGAIGNE.

SOME months since, M. Malgaigne published, in the "Bulletin de Thérapeutique," an account of a method which he practised successfully for the reduction of dislocations of the os humeri. The following is an instance of the total failure of the old method, and the success of the new:

A stone-cutter, forty-one years of age, fell from a scaffold four feet high, and dislocated his shoulder. A surgeon, who saw him immediately after the accident, having made some attempts, pronounced that the reduction was accomplished. After some time, however, the patient, finding he did not regain the use of his joint, entered the hospital Saint Louis, under the care of M. Jobert. A dislocation of the os humeri into the axilla was easily recognized—now twenty-three days after the accident. M. Jobert proceeded to attempt the reduction by the usual method of extension and counter-extension; the arm carried in the horizontal direction. The first, second, and third attempts were equally futile, the pain increasing in proportion to the efforts at reduction. The method of M. Malgaigne was then adopted. An assistant stood upon a table close to the seat of the patient, placed his foot upon the left shoulder to make counter-extension, and pulled with his two hands the dislocated arm raised to a nearly vertical direction. The reduction took place immediately almost without effort, and, above all, with very little pain. M. M. reports a case in which he succeeded after twenty-one days. These cases seem to point out the truth of his assertion, that the cicatrization of the broken ligaments, and the formation of the new capsule, is not perfectly finished till the expiration of twenty-five or thirty days.

*Bulletin Général de Thérapeutique. Avril*, 1838.

---

*On a new Method of distinguishing Cataract.* By M. SANSON.

IF a light is held before an amaurotic eye, the pupil of which is dilated, either in consequence of the disease or by the action of belladonna, three images of the flame are always distinctly visible. Two of these are upright; the third is inverted. The images are situated in the following order. The one in front is upright and the brightest; the one behind is the palest, and is also upright; the third, which is situated between these, is inverted. If the light is moved from side to side, or round and round, the inverted image moves also, but always to the side opposite to the light, whilst the other images follow a uniform course in relation to the light. Hence, to observe these images, the light must be carried in various directions, observing the same positions. These three images are always seen, provided there is no disturbance of the crystalline apparatus: but, whatever may be the degree of the disease, they are not seen when cataract exists. In several cases, where patients were supposed to have had cataract, the three images were seen on employing the light; and it was determined by subsequent examination, that they were either affected with glaucoma or amaurosis.

Experiments were made to determine the causes of these reflected images; together with those on which depend the changes in their number and position. If a light is placed before the convex surface of a watch-glass, an upright image of the flame is seen. If several such glasses are placed, one over the other, the number of upright images corresponds with the number of glasses. Now, in the eye, there are two superimposed convex surfaces: 1, the cornea; 2, the anterior capsule of the crystalline lens. Thus, the two upright images are accounted for. On the other hand, if a light is placed before the concave surface of a watch-glass, an inverted image is seen. If afterwards, another glass is placed in front of it, so as to form a convex lens, two images become visible; one upright, the other inverted. But, conformably to certain physical laws, the inverted image is situated anterior to the upright one. This remark explains the situation of the third image observed by M. Sanson, and which is consequently produced by the concave surface of the posterior capsule of the crystalline lens.

It is now easy to understand how the opacity of a portion of the crystalline apparatus must destroy this phenomenon. Experiments have been made by some medical men, in order to ascertain if the absence of one or more of the images could serve as a means of diagnosticating the seat of cataract, in any particular point of the crystalline lens. These researches have not yet been conducted to any certain results; but several cases have already been witnessed, where the signs above mentioned have enabled M. Sanson to recognize amaurosis mistaken for cataract; and others, in which the absence of these images has led to the inference that cataract existed where the disease was supposed to be simply amaurotic.

[If the above observations are confirmed by the experience of others, and their application is as simple as it is beautiful, there are many cases in which they may materially assist those whose opportunities of examining diseased eyes are not very frequent, in arriving at a correct diagnosis. Sir David Brewster long ago pointed out the use of catoptrically examining the eye in diseases of the cornea. Holding a candle about fifteen inches from the eye, he observed the variations in the size and form of the image of the candles, reflected from the cornea, as he made the candle pass in different directions; and in this way detected the irregularities in conical cornea. The observations of M. Sanson afford a most important extension of the same principle.]

*Archives Générales de Médecine. Décembre,* 1837.

---

*On the Development of Adhesions of Serous Membranes, and their Application to several Surgical Diseases.* By M. Belmas.

This essay was read to the French Institute. M. Belmas quoted a case of spontaneous cure of a hernia, by means of a false membrane obliterating the hernial opening. He afterwards endeavoured to effect, by art, an imitation of this spontaneous cure; and, in his communication, he asserts that he has ascertained the method of developing, at pleasure, adhesions of serous membranes, determining their nature and regulating their extent!

The first experiments were made by means of small bladders of gold-beater's skin, filled with air, and fixed by a small metallic tube in some part of the internal parietes of the abdomen of certain animals: but, as the necks of these bladders did not afford sufficient resistance to the action of the intestines, they were constantly carried away from the points of their insertion. This mode of operating was therefore abandoned; although it served as the basis of a procedure by which several cases of hydrocele were cured. In order to observe the effects produced by the presence of the above bladders of gold-beater's skin, and to prevent their rupture, they were inserted loose into the abdominal cavity of several dogs. The definitive result was, the absorption of the foreign body and adhesion of the parts which it occupied. In order to apply this fact to the obliteration of the neck of the hernial sac, after having made many experiments upon dogs affected with hernia, M. Belmas operated several times on the human subject. The observation of the results having shown him that the quantity of animal matter was far too considerable, he tried the action of simple filaments of dried gelatin, covered by small pieces of gold-beater's skin. Having now ascertained the means of developing linear adhesions between serous surfaces by means of this new agent, he inserted these filaments, by a needle constructed for the purpose, into the neck of the hernial sac. Five cures have been effected out of ten cases, to which this mode of operating has been applied. In three cases there was partial relapse, and in two the disease speedily returned: but, adds the author, as not one of these attempts was attended by the slightest accident, it is to be hoped that more dexterity in the performance of the operation, and a more regular compression upon the ring by means of well-adapted compressing apparatus, will lead to more satisfactory results.

[The facts contained in this communication (although we lament that the journal from which they are extracted has not entered into a little useful detail of the steps, duration, &c. of the operation in the ten cases alluded to,) seem to establish the conclusion that, by the introduction of some foreign body, a sufficient amount of adhesive inflammation may be safely excited on the serous surface of hernial sacs to prevent the reescape of their contents from the abdominal cavity. If the gelatine filaments employed by M. Belmas are equally efficacious, they appear to be far preferable to the double row of pins recommended by M. Bonnet for the same purpose.]

*Revue Médicale française et étrangère. Décembre,* 1837.

---

*Reduction of an old Luxation of the Elbow.* By M. MALGAIGNE.

M. MALGAIGNE has lately reduced, with M. Lisfranc, a luxation of the elbow, backwards, which had existed three months and twenty-one days, in a child ten years of age. "The nature and age of the luxation in so young a child were probably the causes of this success," says M. Malgaigne; "a fact without a parallel in the history of surgery, and one which should reassure the surgeon against the fear of breaking the epiphyses, when he makes use of an appropriate apparatus." These two surgeons employed direct traction with pulleys, and at one time carried it to a force of 300 pounds. The reduction was afterwards accomplished by a novel procedure, which consisted in drawing the arm and fore-arm backwards, whilst the olecranon was pushed with the knee gently forwards and downwards.

*Ibid. Décembre,* 1837.

---

*Ligature of the Carotid Artery of a Child aged Fifteen Months, for the Cure of a Vascular Nævus.* By Dr. ZEIS, of Dresden.

A. D. was born with a vascular nævus on the left cheek, which gradually increased in size, notwithstanding the employment of various remedies; and when seen by Dr. Zeis, in the fifteenth month, extended anteriorly across the cheek to within a short distance of the angle of the mouth, upwards as high as the zygoma, and backwards behind the ear involving the lobe. The central and more elevated part exhibited enormous dilatation of the capillary vessels, some of which were of a bright red, others of a deep blue colour. The child's general health was good, with the exception of some irritation produced by dentition.

The extirpation of the tumour presented so much difficulty and danger, that Dr. Zeis determined on tying the carotid artery, and having given a full trial to astringents, without any satisfactory result, he proceeded to the operation, which was performed on the 30th August. The different steps of the operation are minutely described; but, although rendered difficult by the confined space, presented nothing remarkable. After much trouble, Dr. Z. succeeded in passing a blunt ligature needle, bent laterally from the handle, under the artery. A portion of cellular membrane which lay over the head of the needle was divided; a strong silk thread introduced, and, having satisfied himself that there was no nerve or other part included, the needle was withdrawn and the ligature knotted.

During the operation the child screamed and lay quietly by turns, and the temperature of the body had somewhat diminished, although the loss of blood amounted only to a few ounces. Towards the end of the operation she began to sob, and when the ligature was tightened, broke out into a violent fit of screaming, during which her voice appeared somewhat altered, and hoarser than usual. The wound was covered with a fold of lint dipped in creosote water, and united by means of strips of adhesive plaister. In the evening, the pulse was 108, with increased thirst, and restlessness alternating with sleep. She was ordered an antifebrile mixture with small doses of calomel, and in a few days everything went on favorably.

For some time after the operation, the little patient was attacked with fits of coughing whenever she swallowed anything; this symptom, however, disappeared after the eighth day. Her respiration was at all times easy and natural, but her voice remained for a long time hoarse and squeaking, and it never regained the strength or clearness of tone which it had possessed before the operation. It was also remarkable that she had a habit of sobbing frequently, which she had never exhibited previously, and for which no cause could be assigned. These phenomena, according to Dr. Z., appeared to show that a nervous twig was included in the ligature.

On the morning of the eighth day, Dr. Z. cut short the ligature applied to the carotid, with the view of allowing the wound to heal; and having been obliged to remove the dressing in the evening, in consequence of its being soaked with blood, he was surprised to find the little loop of the ligature lying in the lower angle of the wound. Its early detachment was in all probability favoured by an attack of vomiting which the child had about this period. After this, the wound filled up rapidly, with the exception of a single point, from which matter continued to be discharged until the 11th of November, when complete cicatrization took place.

Immediately after the ligature was tied, the tumour collapsed greatly, but began to swell a little again in the evening, when the temperature of the body rose. It afterwards gradually increased, and in a few weeks attained a very considerable volume, but never exceeded its original size, and the portion behind the ear entirely disappeared. On the 3d of November, a few days before the wound was completely cicatrized, the child was suddenly attacked with convulsions, and hemiplegia of the *right* side. These symptoms were followed by increasing debility, emaciation, and exhausting perspirations, and the little patient died on the 22d of December, sixteen weeks after the operation. For several days before death, she laboured under tonic spasms alternately of the flexor and extensor muscles of the right extremities. The nævus had completely disappeared, leaving behind only a few loose folds of skin. The parents would not permit an examination of the body.

*Zeitschrift für die gesammte Medicin*, iii. Band; i. Heft.

---

*Case of Abscess beneath the Pectoralis Major, communicating with the Cavity of the Chest.* By M. Boinet.

This was the case of a young woman, æt. 15, who was received into the Hôtel Dieu, under the care of M. Blandin. Having severe pain on the right side of the chest, she was supposed to have pleurisy, and was treated accordingly. In a few days considerable tumefaction occurred in the axilla and fore part of the chest, and the presence of pus being evident, an incision was made, from which a large quantity of pus escaped in jets during expiration, whilst during inspiration air entered with a hissing sound: at the same time, a remarkable gurgling sound was heard within the chest, even at the distance of two feet from the patient. A silver catheter, introduced into the incision, readily entered the cavity of the chest to the depth of five or six inches. This communication, however, could not be discovered on the following days. The symptoms were at first somewhat ameliorated after the opening of the abscess, but they soon became worse, and the patient died about ten days afterwards. Percussion had, during life, indicated a clear sound in front to the distance of two inches and a half from the sternum; externally to this, on the side of the chest, there was complete dulness at the level of the sixth and seventh ribs, which gradually diminished both above and below. The respiratory murmur was but little affected. On the left side, the sound from percussion, as well as the respiration, were natural.

*Dissection.* Between the pectoralis major and the eight first ribs is a large cavity extending backwards into the axilla: it contains no pus, and presents a dry and blackish appearance. In the intercostal spaces, between the second, third, and fourth ribs, are nine or ten fistulous openings communicating with the chest; the largest about the size of a fourpenny piece. There are recent but firm adhesions between the lung and the ribs, separating the right side of the chest into two cavities, which communicate with each other by an opening capable of admitting the finger, and through which it is probable that the catheter passed on the day that the abscess was opened. The costal surface of the lung is covered with a thick false membrane. The pulmonary tissue is gorged, but is firm, and does not appear inflamed. The left lung is perfectly healthy, and the other viscera do not present any remarkable pathological appearance.

*Gazette Médicale. May,* 1837.

---

*Successful Application of Creosote in an old Ulcer of the Vagina.*

A female had, in 1833, a syphilitic ulcer of the vagina, which was treated with mercury. The ulcer healed, excepting a part of it on one side of, and beneath, the orifice of the urethra. Again she was mercurialized. In 1834, she was treated by Dr. Bürkner: the ulcer was about the size of a penny, and a quarter of an inch in depth. Regarding it as an atonic fistulous ulcer, it was treated according to the usual rules in such cases, but without effecting any change in it. Creosote was then employed, and applied, by means of charpie, directly to the sore. At first it was used twice, and subsequently once daily. The secretion of pus immediately improved and cicatrization commenced, whilst granulations were formed in the depth of the sore: the patient was completely healed in four weeks.

*Ibid.* No. 36. 1837.

